# Application of HDR-CRISPR/Cas9 and Erythrocyte Binding for Rapid Generation of Recombinant Turkey Herpesvirus-Vectored Avian Influenza Virus Vaccines

**DOI:** 10.3390/vaccines7040192

**Published:** 2019-11-22

**Authors:** Pengxiang Chang, Faisal Ameen, Joshua E. Sealy, Jean-Remy Sadeyen, Sushant Bhat, Yongqing Li, Munir Iqbal

**Affiliations:** 1 Avian Influenza Group, The Pirbright Institute, Pirbright, Woking GU24 0NF, UK; pengxiang.chang@pirbright.ac.uk (P.C.); joshua.sealy@pirbright.ac.uk (J.E.S.); jean-remy.sadeyen@pirbright.ac.uk (J.-R.S.); sushant.bhat@pirbright.ac.uk (S.B.); 2 Department of Microbiology, University of Veterinary and Animal Sciences Lahore 54000, Pakistan; faisal2u1@hotmail.com; 3 Institute of Animal Husbandry and Veterinary Medicine, Beijing Academy of Agricultural and Forestry Sciences, Beijing 100097, China; chunyudady@sina.com; 4 Sino–UK Joint Laboratory for the Prevention & Control of Infectious Diseases in Livestock and Poultry, Institute of Animal Husbandry and Veterinary Medicine, Beijing 100097, China

**Keywords:** herpesvirus of turkeys (HVT), avian influenza, CRISPR/Cas9, HDR

## Abstract

Avian influenza viruses (AIVs) are highly contagious and have caused huge economical loss to the poultry industry. AIV vaccines remain one of the most effective methods of controlling this disease. Turkey herpesvirus (HVT) is a commonly used live attenuated vaccine against Marek’s disease; it has also been used as a viral vector for recombinant AIV vaccine development. The clustered regularly interspaced palindromic repeats (CRISPR)/Cas9 system is a gene editing tool which, in vaccinology, has facilitated the development of recombinant DNA viral-vectored vaccines. Here, we utilize homology-directed repair (HDR) for the generation of a HVT–H7N9 HA bivalent vaccine; a H7N9 HA expression cassette was inserted into the intergenic region between UL45 and UL46 of HVT. To optimize the selection efficiency of our bivalent vaccine, we combined CRISPR/Cas9 with erythrocyte binding to rapidly generate recombinant HVT–H7HA candidate vaccines.

## 1. Introduction

Influenza A viruses are enveloped negative-sense single-stranded RNA viruses with a broad host range, which includes wild aquatic birds that are the natural reservoir for the majority of the virus subtypes [1]. The surface glycoprotein hemagglutination (HA) is the primary target for neutralizing antibodies; however, due to a high polymerase error rate during virus replication, influenza viruses rapidly accumulate mutations that facilitate immune escape from vaccine-induced immunity, which results in vaccine failure. Therefore, vaccines must be updated regularly to be effective. The most frequently used vaccines in the poultry industry are inactivated whole-virus vaccines, and this often includes Fowlpox virus- or Newcastle disease virus (NDV)-vectored vaccines. The efficacy of these vaccines is severely impaired by the presence of maternal antibodies within target hosts [2,3]. Furthermore, the virus-vectored HA vaccine only elicits antibody against viral HA protein, hence can be easily differentiated from the infected birds. However, the inactivated whole-virus vaccines induce antibodies against all viral proteins, which makes it difficult to differentiate infected from vaccinated birds. Therefore, a virus-vectored avian influenza vaccine that is efficacious in the presence of maternal antibodies is highly demanded.

Turkey herpesvirus (HVT) is a naturally nonpathogenic alphaherpesvirus that was originally isolated from turkeys [4]. It has been widely used as live vaccine to control Marek’s disease (MD) in chickens and has demonstrated life-long protection even in the presence of maternal antibodies [5]. HVT vaccines can be administrated either on the day of hatch or in ovo at day 18 of embryonation [6]. Furthermore, in ovo vaccination typically utilizes automation, which significantly improves the speed and ease of vaccination, making in ovo vaccination a preferred route for many poultry industries. HVT has a large double-stranded DNA genome and a narrow host range; therefore, HVT has been exploited for use as a virus vector for expressing a range of antigens from other important avian pathogens. Such antigens include: HA of avian influenza virus (AIV), fusion (F) of NDV, glycoprotein D (gD) and gI of infectious laryngotracheitis virus, and viral protein 2 of infectious bursal disease virus [7,8,9,10]. Recombinant bivalent HVT-vectored vaccines demonstrate robust and long-lasting protection against both the intended diseases, e.g., MDV and NDV [8]. Several nonessential sites, such as the intergenic region between unique long (UL) 45 and UL46, and unique short (US) 2 and US10, are identified in HVT genome that are compatible for foreign gene insertion [10,11,12]. Among these sites, the intergenic region between UL45/46 has been frequently used as an insertion site [7,13,14].

Several recombinant HVT-vectored vaccines have been generated expressing HA of H5N1, H7N1, or H9N2 AIV, and their efficacy has been investigated [7,15,16]. Among these studies, the HVT vector AIV H5HA vaccine, (Vectormune^®^ AI, CEVA Animal Health, Lenexa, KS, USA), is commercially available. It has been demonstrated to be effective in preventing the development of the clinical disease and suppressing virus shedding in vaccinated chickens when challenged with heterologous highly pathogenic H5 AIV [17]. Furthermore, the Vectormune^®^ AI also can provide protection for turkeys and domestic ducks against highly pathogenic H5 AIV [18,19]. It was shown that recombinant HVT–H7HA could protect chickens from lethal challenge against highly pathogenic H7N1 and very virulent MDV, despite low H7HA-specific antibody responses [7]. Recently, a study by Liu et al. showed that recombinant HVT expressing H9HA could induce strong humoral and cellular immune responses and completely abolish H9N2 AIV shedding and transmission in vaccinated chickens [15].

Several genome modification methods have been employed in the past to generate recombinant HVT vaccines, such as homologous recombination, fosmid system construction, and bacterial artificial chromosome (BAC) [20,21,22]. Often, these methods are time-consuming and labor-intensive. Clustered regularly interspaced palindromic repeats (CRISPR)/associated Cas9 technology has gained popularity in recent years for its versatility and specificity in gene editing [23]. Both homology directed repair (HDR)- and error-prone non-homologous end joining (NHEJ)-dependent CRISPR/Cas9 methods have been used to generate recombinant vaccines, with improved speed and efficiency [14,24,25]. Although NHEJ-dependent CRISPR/Cas9 is efficient and easy to use, it leads to bi-directional knock-in of genes, and unintended insertion or deletion of nucleotides near the insertion junction [24]. HDR-dependent CRISPR/Cas9 provides high-fidelity and has been applied in precision genome editing. Thus, in this study, we investigate HDR-dependent CRISPR/Cas9 as a tool for generating HVT–AIV bivalent vaccines. 

## 2. Materials and Methods

### 2.1. Virus, Cell, and Transfection

The turkey herpesvirus FC126 strain was obtained from Avian Disease and Oncology Laboratory (ADOL) East Lansing, MI, USA. The virus stock was propagated in primary chick embryo fibroblast (CEF) cells and kept in liquid nitrogen. The CEF cells were maintained with Dulbecco’s Modified Eagle’s medium (DMEM) supplemented with 5% fetal calf serum (FCS) (Sigma-Aldrich, St. Louis, MO, USA), 10% tryptose phosphate broth solution (Gibco, Life Technologies Ltd., Paisley, UK), 100 U/mL penicillin, and 100 μg/mL streptomycin (Gibco) at 37 °C under a 5% CO_2_ atmosphere. CEF cells were transfected using TransIT-X2^®^ according to the manufacture’s protocol (Mirus, Cambridge Bioscience, Cambridge, UK).

### 2.2. Construction of gRNA and Donor Plasmids

The guide RNA (gRNA) against the UL45/46 intergenic region of HVT and enhanced green fluorescent protein (eGFP) was adapted from a previous publication [26]. The DNA oligo of gRNA was synthesized (Sigma-Aldrich, St. Louis, MO, USA) and cloned into plasmid pX459-v2 (Addgene, Cambridge, MA, USA) using BbsI cloning site. To construct the donor plasmid, the complete coding region of H7N9 HA (A/Guangdong/SF003/2016) or eGFP was amplified using gene specific primers listed in Table 1 and cloned into plasmid pHVT-UL45-46 using KpnI/XbaI restriction sites with Pec promoter (CMV enhancer and chicken β-actin chimera promoter) at the 5′ end and SV40 polyadenylation (poly A) at the 3′ end [7]. The resulting plasmids were termed donor H7HA or donor GFP respectively.

### 2.3. HDR-CRISPR/Cas9-Mediated Gene Insertion

CEF cells were transfected with 0.5 µg gRNA plasmids and 0.5 µg donor plasmids per well of 12-well plates before infection with HVT at different multiplicity of infection (MOI). Virus was harvested at 72 h post-infection and subjected to plaque purification.

### 2.4. HVT Genome Extraction

CEF cells infected with HVT were harvested at 72 h post-infection and lysed in quishing buffer (10 mM Tris-HCl, pH 8, 1mM EDTA, 25 mM NaCl, and 200 µg/mL Proteinase K) at 65 °C for 30 min, and the reaction was terminated by heating at 95 °C for 5 min.

### 2.5. Chicken Red Blood Cell Hemadsorption Assay

We developed hemadsorption assays for detection of HA antigen hemagglutination activity, in which HA antigen expressed by rHVT specifically adsorbed the chicken red blood cells (RBCs). The RBC adsorption at the rHVT–HA expressing plaque can be visualized directly by eyes within 30 min. Briefly, CEF cells were infected with HVT for 72 h. The CEF cells were then washed twice with phosphate-buffered saline (PBS) before addition of 2 mL of 0.5% chicken red blood cells per well of 6-well plates. Plates were kept at room temperature for 30 min before the non-adsorbed red blood cells were aspirated and plates replenished with PBS.

### 2.6. Immunostaining Assays for H7 HA Antigen Detection

CEF cells were infected with HVT–H7HA for 72 h and then immunohistochemical stained as described previously [24]. The expression of H7N9 HA antigen in HVT–AIV vaccine-infected cells was visualized by incubating cells with AIV H7N9 HA mouse monoclonal antibody (generated at the Pirbright Institute and used at 1/200 dilution), followed by horseradish peroxidase-conjugated rabbit anti-mouse secondary antibody (used with 1/200 dilution) (DAKO, Agilent Technologies, Santa Clara, CA, USA). The staining was developed by with 3,3′-diaminobenzidine (DAB) substrate-chromogen solution (DAKO). The images were taken using the Leica fluorescence microscope (Leica, Wetzlar, Germany).

### 2.7. Indirect Immunofluorescence Assay (IFA)

CEF cells grown on the coverslip in 24-well plates were infected with HVT or HVT–H7HA at MOI 0.001 or mock-infected for 48 h. The cells were washed with PBS and then fixed with 4% paraformaldehyde and permeabilized with 0.1% Triton X-100 (Sigma-Aldrich, St. Louis, MO, USA) in PBS. The cells were blocked with 1% bovine serum albumin (BSA) (Sigma-Aldrich, St. Louis, MO, USA) before being probed with H7N9 HA mouse monoclonal antibody (used at 1/200 dilution) and chicken anti-HVT antiserum (generated at the Pirbright Institute and used at 1/200 dilution). The cells were then incubated with Alexa Fluor 568-labeled anti-mouse and Alexa Fluor 488-labeled anti-chicken secondary antibodies at 1/200 dilution (Invitrogen, Life Technologies Ltd., Paisley, UK). The cover slips were mounted with Vectashield mounting medium with 4′,6-diamidino-2-phenylindole (DAPI) (Vector Laboratories, Burlingame, CA, USA). The images were taken using the Leica TCS SP5 confocal laser scanning microscope (Leica, Wetzlar, Germany).

### 2.8. Western Blot

CEF cells infected with HVT or HVT–H7HA at MOI 0.1 for 72 h were lysed using Radioimmunoprecipitation Assay (RIPA) Lysis and Extraction Buffer (Life Technologies Ltd., Paisley, UK). Chicken antiserum raised against H7N9 HA (used at 1/1000 dilution) and rabbit polyclonal anti-α-Tubulin (1 in 3000 dilution) (Abcam, Cambridge, MA, UK) were used to detect HA and α-Tubulin proteins, respectively. Secondary anti-mouse or anti-rabbit IgG antibodies (1 in 10,000 dilution) labeled with florescent dyes IRDye 800CW or IRDye 680RD (Li-COR, Lincoln, Nebraska, USA), respectively, were visualized using the Odyssey CLx (Li-COR).

### 2.9. Statistical Analysis

**Figure 1:**
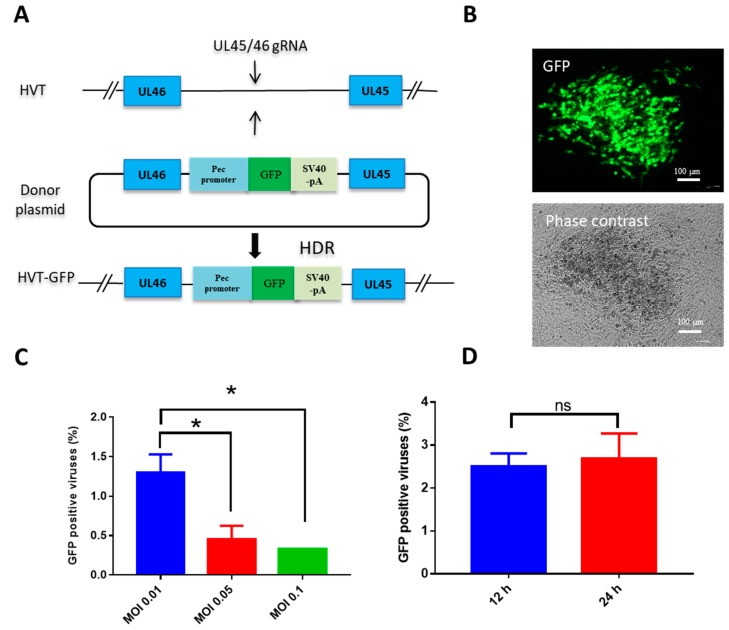
Optimization of gene knock-in via CRISPR/Cas9-induced homology-directed repair (HDR) repair. (**A**) Schematic of insertion of green fluorescent protein (GFP) expression cassette between turkey herpesvirus (HVT) UL45 and UL46; (**B**) HVT–GFP plaque under UV excitation or phase contrast; (**C**) the efficiency of GFP cassette knock-in with different HVT infection doses; (**D**) the efficiency of GFP cassette knock-in with different infection times post-transfection. Data shown represent the mean of three replicates. One-way ANOVA (Figure 1C) and paired Student’s *t*-test (Figure 1D) were used to test differences between different groups. Error bar = standard error of mean. * *p* < 0.05; ns = not significant; MOI = multiplicity of infection.

Statistical analysis was performed using GraphPad Prism 7 (GraphPad Software, La Jolla, CA, USA). One-way ANOVA (Figure 1C) and paired Student’s *t*-test (Figure 1D) were used to test differences between different groups. *p*-values <0.05 were considered significant.

## 3. Results

### 3.1. Optimization of HDR-CRISPR/Cas9 for Gene Knock-in to HVT 

To investigate the potential for HDR-CRISPR/Cas9 to be used as a tool for knocking a gene into the HVT genome, a GFP expression cassette was selected to insert into the intergenic region between UL45/46 (Figure 1A). We adopted transfection and infection methods to generate recombinant HVT as previously reported [14]. Both virus dose and the time of virus infection post-transfection affect the efficiency of recombination [27]. Therefore, to determine the optimal virus dose to be used in gene knock-in, CEF cells were transfected with HVT gRNA and donor GFP plasmids, and were infected with HVT at MOI 0.01, 0.05, and 0.1 at 12 h post-transfection, respectively. The MOI 0.01 yielded the most efficient GFP knock-in, at ~1.0%, as evidenced by GFP positive colonies (Figure 1B,C). Efficiency reduced when higher infection doses of MOI 0.05 and 0.1 of HVT were applied, and there was no difference in knock-in efficiency between infection at 12 h and 24 h post-transfection (Figure 1D).

### 3.2. HDR-CRISPR/Cas9 Knock-in of H7N9 HA into HVT

**Figure 2:**
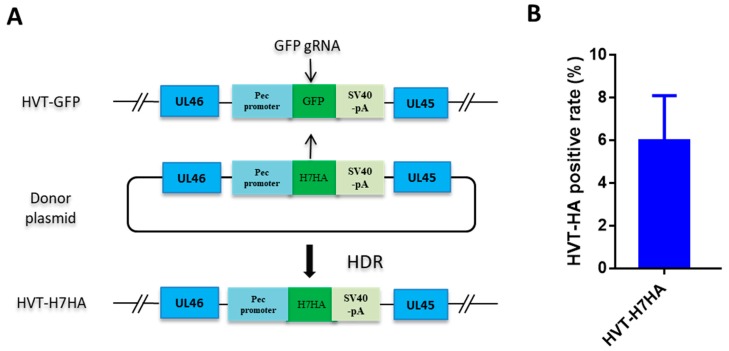
Recombinant HVT–H7HA generation via HDR-CRISPR/Cas9. (**A**) Schematic depicting the use of HDR-CRISPR/Cas9 in the construction of recombinant HVT–H7HA. The GFP expression cassette was replaced by H7HA expression cassette via HDR-CRISPR/Cas9 with gRNA- targeted GFP, the plaques formed by HVT infected cells without green fluorescence were isolated, and then subjected to PCR screening using primers targeting the intergenic region between UL45 and UL46. (**B**) The efficacy of HVT–H7HA gene recombination. The indicated percentage of HVT–H7HA positive plaques were calculated from the total number of 24 HVT plaques without showing green fluorescence. The results are the average of three independent repeats. Error bar  =  standard error of mean.

After optimizing conditions for GFP knock-in using HDR-CRISPR/Cas9, we proceeded to knock-in an influenza H7N9 HA expression cassette into the HVT genome for the development of a bivalent vaccine against both Marek’s disease and H7N9 avian influenza. Typically, screening of recombinant HVT is conducted by including a fluorescent marker, along with the antigen expression cassette, which later gets removed using Cre recombinase enzyme [14]. However, this methodology involves a time-consuming two rounds of plaque purification; in the first round, HVT–antigen plaques with GFP tag are purified, and in the second-round, plaques of HVT–antigen only are purified after the removal of GFP. To expedite the isolation of recombinant HVT–H7HA, we replaced the GFP cassette of HVT–GFP with a H7HA expression cassette using HDR-CRISPR/Cas9, whereby plaques formed by HVT-infected cells without green fluorescence were isolated, as they likely contained HVT–H7HA (Figure 2A). The isolated individual clones of rHVT–H7HA viruses were then expanded, and viral DNA was extracted and subjected to PCR analysis using primers targeting the intergenic region between UL45 and UL46 (Table 2). In total, ~6% of clones were positive for the H7N9 HA insertion (Figure 2B).

### 3.3. Selection of Recombinant HVT–H7HA by Erythrocyte Binding

**Figure 3:**
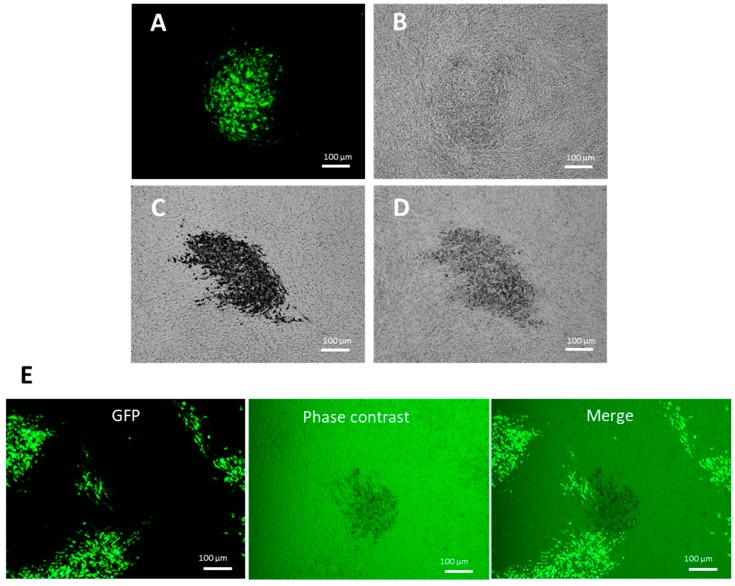
Selection of recombinant HVT–H7HA using chicken erythrocyte binding. Chick embryo fibroblast (CEF) cells were infected with HVT–GFP, HVT–H7HA, or unpurified HVT–H7HA and then subjected to chicken erythrocyte binding. CEF cells infected with HVT–GFP under UV excitation (**A**) and phase contrast (**B**). CEF cells infected with HVT–H7HA and then immunohistochemistry stained using mouse monoclonal antibody against H7HA (**C**) or with chicken erythrocyte binding (**D**). (**E**) CEF cells infected with unpurified HVT–H7HA under UV excitation, phase contrast, and a merge of both.

Influenza virus glycoprotein HA binds to cellular receptors that are present on the surface of erythrocytes, which adsorb onto the cells. This forms the basis of the virus hemagglutination assay, whereby virus–erythrocyte binding forms a lattice that prevents erythrocytes from settling out of suspension and forming a characteristic “button” at the bottom of a v-bottom well [29]. To investigate whether erythrocytes can be adsorbed to HA protein expressed by the CEF cells infected with recombinant HVT–H7HA, we added 0.5% chicken red blood cells (RBCs) to the CEF cells infected with recombinant HVT–GFP and HVT–H7HA. After 30 min of incubation, RBCs were removed, and cells were replenished with phosphate-buffered saline (PBS). Recombinant HVT–GFP was confirmed by green fluorescence under UV excitation, and recombinant HVT–H7HA was confirmed by immunochemistry staining using H7N9 HA specific monoclonal mouse antibodies (Figure 3A,C). In sharp contrast to the recombinant HVT–GFP viruses (Figure 3B), the plaques of recombinant HVT–H7HA showed strong adsorption to RBCs under phase contrast microscope (Figure 3D). These results demonstrate that RBCs can specifically bind to CEF cells infected with recombinant HVT–H7HA. To test whether this can be exploited for recombinant HVT–H7HA selection, the unpurified recombinant HVT–H7HA virus generated by HDR-CRISPR/Cas9 was used to infect CEF cells, followed by RBC adsorption assay. Recombinant HVT–H7HA positive plaques were clearly distinguishable, showing RBC adsorption through phase contrast (Figure 3E).

### 3.4. Characterization of Recombinant HVT–H7HA

**Figure 4:**
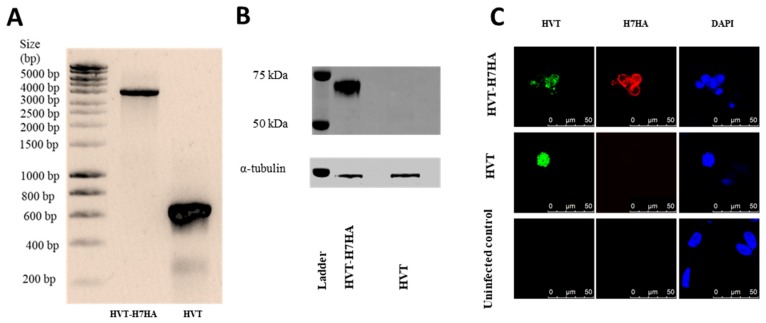
Characterization of HVT–H7HA. (**A**) PCR products of H7HA antigen cassette inserted between UL45 and UL46; (**B**) Western blot detection of H7HA protein from HVT–H7HA-infected CEF cells. Comparable protein loading in each lane was demonstrated by α-Tubulin detection. (**C**) Analysis of HA expression by indirect immunofluorescence assay. The H7HA was detected by staining with H7HA-specific mouse monoclonal antibody, followed by Alexa Fluor 568-labeled anti-mouse secondary antibody (red fluorescence). The HVT was detected by staining with HVT chicken antiserum, followed by Alexa Fluor 488-labeled anti-chicken secondary antibody (green fluorescence). The nuclei were stained with DAPI (blue fluorescence).

Next, the insertion of the H7N9 HA cassette within the HVT genome between UL45/46 site was assessed. PCR amplification of the H7HA antigen cassette from HVT–H7HA using primers from Table 2 showed an expected product size of ~3900 bp, while control wild-type HVT with no cassette showed an expected product size of ~600 bp (Figure 4A). The expression of H7HA protein was confirmed by Western blot using chicken antiserum raised against H7N9 virus, with α-Tubulin as a protein loading control (Figure 4B). The cell lysates from CEF infected with HVT–H7HA showed a ~70 kDa HA protein, while no detectable HA protein signal was observed with lysates of cells infected with control wild-type HVT. The expression of H7HA was then determined by indirect immunofluorescence assay (IFA) (Figure 4C). As expected, cells infected with HVT–H7HA showed clear double staining with both H7HA (red) and HVT (green) positive staining. Cells infected with wild-type HVT showed only HVT positive staining (green), while no HA and HVT staining was observed in control uninfected cells.

## 4. Discussion

HDR-CRISPR/Cas9 is a powerful tool for genome engineering, with potential in many areas of biological science. In this study, we have, for the first time, utilized HDR-CRISPR/Cas9 in a transfection and infection approach to generate a recombinant bivalent HVT vaccine expressing a major influenza A virus antigen, hemagglutinin. To aid the recombinant HVT–HA selection, we developed an erythrocyte adsorption assay to precisely, easily, and quickly identify recombinant HVT virus plaques in live cultured cells expressing influenza virus hemagglutinin glycoprotein.

In our experience, the bacterial artificial chromosome (BAC) system for recombinant HVT–HA (H7N1) vaccine generation is comparably time-consuming [7]. It takes ~two weeks to insert a HA expression cassette into the HVT-BAC system, and a further three weeks to remove the BAC for the HVT–HA vaccine generation, whereas using the method described in this study, it only takes two and a half weeks to generate an HVT–HA vaccine seed strain, thus saving two and a half weeks compared to the use of BAC. Moreover, the HVT-BAC-HA system is often unstable in bacteria, making it difficult to generate recombinant HVT viruses. In comparison, CRISPR/Cas9 acts on the virus genome directly, meaning no bacterial work is required. In recent years, both NHEJ and HDR-dependent CRISPR/Cas9 have been used for recombinant vaccine development. Previously, Tang et al. used NHEJ-CRISPR/Cas9 to insert a VP2 expression cassette into the genome of HVT, and demonstrated significant convenience and efficiency [14]. When used alongside CRISPR/Cas9, NHEJ is considered to have greater efficiency than HDR because genome repair occurs throughout the cell cycle, while HDR only occurs during S and G2 phases of the cells life cycle [30]. Another attractive trait of NHEJ is that the donor plasmid is free from the restriction of homology arms, and is therefore beneficial for the donor plasmids shared between different virus vectors or different insertion sites inside one virus vector. However, the downside of NHEJ is that small insertion or deletion of nucleotides may occur and the insert is bi-directional [24]. HDR has advantage over these aspects, because of high fidelity and insertion of a cassette in a uni-directional manner. Having high fidelity is particularly important for virus-vectored vaccines, because even small levels of insertion or deletion may alter the stability of the recombinant viruses.

Instead of using fluorescent markers as an indicator for antigen gene incorporation, we used GFP gRNA-mediated HDR-CRISPR/Cas9 to replace the GFP gene with the HA gene, and then isolated plaques without green fluorescence. Plaques without green fluorescence are likely to be clones, either with a silenced GFP due to a disturbed opening reading frame, or recombinant HVT–H7HA due to successful replacement of the GFP gene with the HA gene. However, HVT–H7HA positive rates only reached ~6%, which indicates the majority of GFP negative HVT viruses are caused by NHEJ-mediated silence. One explanation is that the majority of CEF cells were transfected with GFP gRNA and no donor plasmid. However, there is evidence to show that knock-in of influenza HA into a different region of HVT using the same strategies and the same GFP gRNA can lead to HVT–HA positive rates of ~50% (unpublished data from personal communication). This indicates that the donor plasmids, in particular, the size of the homologous arm, might play an important role, though the exact reason warrants further investigation.

To our knowledge, this is the first study that utilizes virus–erythrocyte binding with phase contrast microscopy to screen for HAs expressed on CEF cells infected with recombinant HVT–HA, thus identifying successfully generated recombinant HVT viruses. Compared with screening methods that use HA-specific monoclonal antibodies tagged with fluorescence protein, erythrocyte-based screening is cheap, fast, and can be applied to recombinant viruses expressing HA of the majority of influenza virus, apart from some recent human influenza virus HA that lost RBC binding ability. This method is also not restricted to recombinant virus expression of influenza HA, but can be potentially applicable for selection of recombinant viruses expressing NDV hemagglutinin-neuraminidase proteins which also adsorb to erythrocytes.

## 5. Conclusions

To conclude, we have demonstrated HDR-CRISPR/Cas9, together with erythrocyte selection, is a powerful and speedy technology in recombinant avian influenza vaccine development. Furthermore, HDR-CRISPR/Cas9 can be used for the development of recombinant vaccines to protect chickens against a wide variety of economically important pathogens, and the erythrocyte-adsorption assay can be used with any target antigen that has erythrocyte adsorption activity.

## Figures and Tables

**Table 1:** Guide RNA.

gRNA	Target Sequence 5′-3′	Gene Locus
HVT gRNA	AAAACACAGTAACCGTTAGAGG	UL45/46
GFP gRNA	AGCTGGACGGCGACGTAAACGG	eGFP

This primer sequences are adapted from published data [28].

**Table 2:** Primer list.

Primer Name	Sequence 5′-3′
**eGFP forward**	ATTATTGGTACCATGGTGAGCAAGGGCGAG
**eGFP reverse**	GCCGCTTCTAGATTACTTGTACAGCTCGTC
**H7N9 HA forward**	ATAGGTACCATGAACACTCAAATCCTG
**H7N9 HA reverse**	AATTCTAGATTATATACAAATAGTGCAC
**UL45/46 forward**	GTCTTCCGGTTAAGGGACAG
**UL45/46 reverse**	CGAACAAGTCGGGAAGTACG
